# Bayesian random local clocks, or one rate to rule them all

**DOI:** 10.1186/1741-7007-8-114

**Published:** 2010-08-31

**Authors:** Alexei J Drummond, Marc A Suchard

**Affiliations:** 1 Allan Wilson Centre for Molecular Ecology and Evolution, University of Auckland, Private Bag 92019, Auckland, New Zealand; 2 Computational Evolution Group, University of Auckland, Private Bag 92019, Auckland, New Zealand; 3 Departments of Biomathematics and Human Genetics, David Geffen School of Medicine at UCLA, Los Angeles, CA 90095, USA; 4 Department of Biostatistics, UCLA School of Public Health, Los Angeles, CA 90095, USA

## Abstract

**Background:**

Relaxed molecular clock models allow divergence time dating and "relaxed phylogenetic" inference, in which a time tree is estimated in the face of unequal rates across lineages. We present a new method for relaxing the assumption of a strict molecular clock using Markov chain Monte Carlo to implement Bayesian modeling averaging over random local molecular clocks. The new method approaches the problem of rate variation among lineages by proposing a series of local molecular clocks, each extending over a subregion of the full phylogeny. Each branch in a phylogeny (subtending a clade) is a possible location for a change of rate from one local clock to a new one. Thus, including both the global molecular clock and the unconstrained model results, there are a total of 2^2*n-*2 ^possible rate models available for averaging with 1, 2, ..., 2*n - *2 different rate categories.

**Results:**

We propose an efficient method to sample this model space while simultaneously estimating the phylogeny. The new method conveniently allows a direct test of the strict molecular clock, in which one rate rules them all, against a large array of alternative local molecular clock models. We illustrate the method's utility on three example data sets involving mammal, primate and influenza evolution. Finally, we explore methods to visualize the complex posterior distribution that results from inference under such models.

**Conclusions:**

The examples suggest that large sequence datasets may only require a small number of local molecular clocks to reconcile their branch lengths with a time scale. All of the analyses described here are implemented in the open access software package BEAST 1.5.4 (http://beast-mcmc.googlecode.com/).

## Background

In 1967, Allan Wilson and his then doctoral student Vincent Sarich described an "evolutionary clock" for albumin proteins and exploited the clock to date the common ancestor of humans and chimpanzees to five million years ago [1]. Given the limited informativeness of these immunological data, this estimate has survived the intervening years remarkably well. This work was the first prominent application of the concept of a molecular clock [2] and, at the time, the result raised extreme controversy, as the commonly held belief advocated that the common ancestor of humans and African apes was much more ancient. In fact, previous authors had argued that there must have been a slowdown of the rate of albumin evolution in African apes and humans to reconcile their great similarity with the presumed antiquity of their common ancestor.

Researchers have grappled with the tension between molecular and non-molecular evidence for evolutionary time scales ever since. Recently, a number of authors [3-7], have advanced "relaxed molecular clock" methods. These methods accommodate variation in the rate of molecular evolution from lineage to lineage. In addition to allowing non-clock-like relationships among sequences related by a phylogeny, modeling rate variation among lineages in a gene tree also enable researchers to incorporate multiple calibration points that may not be consistent with a strict molecular clock. These calibration points can be associated either with the internal nodes of the tree or the sampled sequences themselves. Furthermore, relaxed molecular clock models appear to fit real data better than either a strict molecular clock or the other extreme of no clock assumption [6]. In spite of these successes, controversy still remains around the particular assumptions underlying some of the popular relaxed molecular clock models currently employed. A number of authors [8-10], argue that changes in the rate of evolution do not necessarily occur smoothly nor on every branch of a gene tree. The alternative expounds that large subtrees share the same underlying rate of evolution and that any variation can be described entirely by the stochastic nature of the evolutionary process. These phylogenetic regions or subtrees of rate homogeneity are separated by changes in the rate of evolution. This alternative model may be especially important for gene trees that have dense taxon sampling, in which case there are potentially many short closely related lineages amongst which there is not reason a priori to assume differences in the underlying rate of substitution.

Local molecular clocks are another alternative to the global molecular clock [11]. A local molecular clock permits different regions in the tree to have different rates, but within each region the rate must be the same. Up until now these models have been difficult to employ because their implementations did not permit the modeling of uncertainty in (1) the phylogenetic tree topology or (2) the phylogenetic positions of the rate changes between the local clock regions. For a model that allows one rate change on a rooted tree there are 2*n − *2 branches on which the rate change can occur. To consider two rate changes, one must consider (2*n − *2) × (2*n − *3) possible rate placements. If each branch can have 0 or 1 rate changes then the total number of local clock models that might be considered is 2^2*n−*2^, where *n *is the number of sequences under study. For even moderate *n *this number of local clock models can not be evaluated exhaustively.

In this paper we employ Markov chain Monte Carlo (MCMC) to investigate a Bayesian random local clock (RLC) model, in which all possible local clock configurations are nested. We implement our method in the BEAST 1.x [12] and BEAST 2 (http://code.google.com/p/beast2/) open software frameworks. The resulting method co-estimates from the sequence data both the phylogenetic tree and the number, magnitude and location of rate changes along the tree. Our method samples a state space that includes the product of all 2^2*n− *2 ^possible local clock models on all possible rooted trees. Because the RLC model includes the possibility of zero rate changes, it also serves to test whether one rate is sufficient to rule all the gene sequences at hand, as was Wilson and Sarich's view of the African primate albumins.

## Methods

### Basic evolutionary model

We begin by considering data **Y**, consisting of aligned molecular sequences of length *S *from *n *taxa. We orientate these data such that we may write **Y **= (**Y**_1_, ..., **Y***_S_*), where **Y***_s _*for *s *= 1, ..., *S *are the *n *homologous characters at each site *s *of the sequence alignment. To model this homology, we follow standard likelihood-based phylogenetic reconstruction practice [13] and assume the data arise from an underlying continuous-time Markov chain (CTMC) process [14] along an unobserved tree ***τ ***. The tree ***τ ***consists of a rooted, bifurcating topology that characterizes the relatedness among the taxa, the generally unknown historical times when lineages diverge in the topology and up to 2*n − *2 rate parameters *r_k _*that relate historical time and expected number of substitutions on each branch *k*. The CTMC process describes the relative rates at which different sequence characters change along the independent branches in ***τ ***. We restrict our attention in this paper to nucleotide substitution processes characterized by either the HKY85 [15] or GTR [16] infinitesimal rate matrices **Λ **and discrete-Gamma distributed across-site rate variation [17] with shape parameter *α*. However, our approach admits any standardly used CTMC for nucleotides, codons or amino acids.

Letting **Φ **= (**Λ**,*α*), we write the data sampling density of site *s *as *P*(**Y***_s_*|**τ**, **Φ**). Felsenstein's peeling/pruning algorithm [18] enables computational efficient calculations of *P*(**Y***_s_*|**τ**,**Φ**). Assuming that sites are independent and identically distributed given (**τ**,**Φ**) yields the complete data likelihood

(1) $$P\left(Y|\tau ,\;\Phi \right)=\prod_{s=1}^{S}P\left(Y_{s}|\tau ,\;\Phi \right).$$

### Branch-specific rate variation

We take the opinion that variation in the rate of molecular evolution is widespread [5,6], but, following Yoder and Yang [11], we assumed that in any given tree there exist a small number of rate changes. This contrasts with most previous Bayesian MCMC relaxed clock models that favor many small or smoothly changing events [3,7,19,20]. In general, the numerous small changes arise as a modeling consequence, and are not necessarily data-driven. Apart from the induced smoothing, some structure remains quite useful; at certain time scales one expects rate changes to be heritable and persist for some time down the subtree extending from the change-point.

#### Model parameterization

We introduce the RLC model that allows for sparse, possibly large-scale changes while maintaining spatial correlation along the tree. We start at the unobserved branch leading to the most recent common ancestor (MRCA) of the tree and define the composite rate *ρ*_MRCA _= 1. Substitutions then occur on each branch *k *= 1, ..., 2*n − *2 below the MRCA with normalized rate

(2) $$\begin{array}{c} r_{k}=c(\rho )\times \rho_{k}\\ =c(\rho )\times \rho_{\text{pa}(k)}\times \phi_{k}, \end{array}$$

where pa(*k*) refers to the parent branch above *k*, branch-specific rate multipliers ***ϕ ***= (*ϕ*_1_,...,*ϕ*_2*n*-2_) and *c*(·) is a normalization constraint that ensures that *r_k _*reflect the expected number of substitutions per unit time. This multiplicative structure on the composite *ρ_k _*= *ρ*_pa(*k*) _× *ϕ_k _*builds up a hierarchy of rate multipliers descending towards the tree's tips.

Allowing all elements in ***ϕ ***to vary independently leads to a completely non-clock-like model with, even worse, far too many free parameters for identifiability with the divergence times in ***τ***. We avoid this problem through specifying a prior *P*(*ϕ*) on the rate multipliers. This prior specifies that only a random number *K *∈ {0,...,2*n*-2} of *ϕ_k _≠ *1 such that *r_k _*do not inherit their ancestors' rate of change but instead mark the start of a new local clock, where *a priori *we believe *K *is small. In effect, we place non-negligible prior probably on *K *= 0, the state in with one rate rules them all. Further, with most *r_k _*= *r*_pa(*k*)_, the prior binds absolute rates equal on branches incident to the same divergence points.

#### Bayesian stochastic search variable selection

To infer which branch-specific rates *r_k _*do or do not inherit their ancestors' rate, we employ ideas from Bayesian stochastic search variable selection (BSSVS) [21]. BSSVS traditionally applies to model selection problems in a linear regression framework. In this framework, the statistician starts with a large number of potential predictors **X**_1_,...,**X***_P _*and asks which among these associate linearly with an *N*-dimensional outcome variable **Y**. For example, the full model becomes **Y **= [**X**_1_,...,**X***_P_*]***β + ϵ***, where *β *is a *P*-dimensional vector of regression coefficients and ***ϵ ***is an *N*-dimensional vector of normally distributed errors with mean **0**. When *β_p _*for *p *= 1,...,*P *differs significantly from 0, **X***_p _*helps predict **Y**, otherwise **X***_p _*contributes little additional information and warrants removal from the model via forcing *β_p_= *0. Given potentially high correlation between the predictors, deterministic model search strategies tend not to find the optimal set of predictors unless one explores all possible subsets. This exploration is generally computationally impractical as there exist 2*^P ^*such subsets and completely fails for *P > N*.

Recent work in BSSVS [22,23] efficiently performs the exploration in two steps. In the first step, the approach augments the model state-space with a set of *P *binary indicator variables ***δ ***= (*δ*_1_,...,*δ_P_*) and imposes a prior *P*(*β*) on the regression coefficients that has expectation **0 **and variance proportional to a *P × P *diagonal matrix with its entries equal to ***δ***. If *δ_P _*= 0, then the prior variance on *β_p _*shrinks to 0 and enforces *β_p _*= 0 in the posterior. In the second step, MCMC explores the joint space of (***δ***, ***β***) simultaneously.

To map BSSVS into the setting of rate variation, let *δ_k _*be the binary indicator that a local clock starts along branch *k*, such that *r_k _*≠ *r*_pa(*k*)_. Conversely, when *δ_k _*= 0, *r_k _*= *r*_pa(*k*) _implying that *ϕ_k _*= 1. So, rate multipliers ***ϕ ***play an analogous role to the regression coefficients in BSSVS. An important difference is that *ϕ_k _*∈ [0,∞) and shrinks to 1, while *β_k _*∈ (-∞,∞) and shrinks to 0, mandating alternative prior formulations.

#### Prior specification

To specify a prior distribution over ***δ ***= (*δ*_1_,...,*δ*_2*n*-2_), we assume that each indicator acts *a priori *as an independent Bernoulli random variable (RV) with small success probability *χ*. The sum of independent Bernoulli RVs yields a Binomial distribution over their sum $K=\sum_{k=1}^{2n-2}\delta_{k}$. In the limit that *K *≪ χ × (2*n*-2), this prior conveniently collapses to

(3) K~Truncated−Poisson (λ),

where λ is the prior expected number of rate changes along the tree ***τ ***. Choosing λ = log2, for example, sets 50% prior probability on the hypothesis of no rate changes.

Completing the RLC prior specification, we assume that all rate multipliers in ***ϕ ***are *a priori *independent and

(4) $$\phi_{k}\sim \text{Gamma}(1/\psi \delta_{k},1/\psi \delta_{k}).$$

When *δ_k _*= 1, then *a priori*, *ϕ_k _*has expectation 1 and variance *ψ*, following in the vein of [24]. However, in light of BSSVS, when *δ_k _*= 0, the prior variance collapses to 0 and *ϕ_k _*= 1.

#### Normalization

To translate between the expected number of substitutions *b_k _*on branch *k *and real clock-time *t_k_*,

(5) $$b_{k}=\mu \times r_{k}\times t_{k},$$

where *μ *is the overall substitution rate. The keen eye may observe that, over the entire tree ***τ ***, the parameterization in Equation (5) again leads to more degrees-of-freedom than are identifiable. We solve this difficulty through a further normalization constraint *c*(·) on ***ρ***. Recall that we wish to measure *μ *in terms of expected substitutions per unit real time, such that

(6) $$\mu =\sum_{k=1}^{2n-2}b_{k}/\sum_{k=1}^{2n-2}t_{k}.$$

To maintain this scaling, we sum Equation (5) over all branches and substitute the result into Equation (6). This eliminates the unknown *μ *and yields

(7) $$\begin{array}{c} \sum_{k=1}^{2n-2}r_{k}t_{k}=c(\rho )\sum_{k=1}^{2n-2}\rho_{k}t_{k}=\sum_{k=1}^{2n-2}t_{k},\\ c(\rho )=\sum_{k}t_{k}/\sum_{k}\rho kt_{k}. \end{array}$$

### Posterior simulation

We take a Bayesian approach to data analysis and draw inference under the RLC model via MCMC. MCMC straightforwardly generates random draws with first-order dependence through the construction of a Markov chain that explores the posterior distribution. Via the Ergodic Theorem, simple tabulation of a chain realization {***θ***^(1)^,...,***θ***^(*L*)^} can provide adequate empirical estimates. To generate a Markov chain using the Metropolis-Hastings algorithm [25,26], one imagines starting at chain step *ℓ *in state ***θ***^(*ℓ*) ^and randomly proposing a new state θ* drawn from an arbitrary distribution with density *q*(·|***θ***^(*ℓ*)^). This arbitrary distribution is commonly called a "transition kernel". Finally the next chain step *ℓ *+ 1 arrives in state

(8) $$\theta^{\left(\ell +1\right)}=\{\begin{array}{ll} \theta^{⋆} & \text{with}\;\text{probability}:\min\left\{1,\frac{P(\theta^{⋆}|Y)}{P(\theta^{\left(\ell \right)}|Y)}\;\;\times \;\;\frac{q(\theta^{\left(\ell \right)}|\theta^{⋆})}{q(\theta^{⋆}|\theta^{\left(\ell \right)})}\right\},\\ \theta^{\left(\ell \right)} & \text{otherwise}. \end{array}$$

The first term in the acceptance probability above is the ratio of posterior densities and the term involving the transition kernel is the Hastings ratio. The beauty of the algorithm is that the posterior densities only appear as a ratio so that intractable normalizing constant cancels out.

#### Transition kernels

We employ standard phylogenetic transition kernels via a Metropolis-within-Gibbs scheme, as implemented in BEAST [12], to travel through most dimensions in the RLC parameter space. What is unique to the RLC model are transition kernels to explore rate multipliers ***ϕ ***and all possible local clock indicator ***δ ***configurations. Since *ϕ_k _*∈ [0,∞) we propose new rates ϕ* component-wise, such that for a uniform randomly selected *k *with *δ_k _*= 1,

(9) $$\begin{array}{c} \phi_{k}^{⋆}=U\phi_{k},\\ U\sim \text{Uniform}\;\left(s_{f},\;\frac{1}{s_{f}}\right), \end{array}$$

where 0 *< s_f _<*1 is a tuning constant and the Hastings ratio is 1/*U *[27].

Transition kernels on ***δ ***are more challenging. One natural way to construct a Markov chain on a bit-vector state space, such as ***δ***, involves selecting one random element *δ_k _*with uniform probability 1/(2*n − *2) and swapping its state $\delta_{k}^{⋆}=1-\delta_{k}$ with probability 1 [14].

At first glance, the transition kernel density *q*(δ*|***δ***) = *q*(***δ***|δ*) = 1/(2*n *- 2) appears symmetric leading to a Hastings ratio of 1. However, this view is flawed. One must recall that we introduced the indicators ***δ ***as a computational convenience. The number of different local clocks *K *over-shadows ***δ ***as our parameter of interest, upon which we place our truncated-Poisson prior *P*(*K*). The correct densities to calculate then become *q*(K*|*K*) and *q*(*K*|K*). Suppose the swapping event above generates 0 *→ *1 so that K* = *K *+ 1.  As *K *approaches 0 the transition kernel finds it more and more difficult to decrease *K *because the kernel is more likely initially to choose a 0 state for swapping. From this perspective, the kernel is definitely not symmetric in the interchange of K* and *K*. Assuming symmetry would lead to upwardly biased estimates for *K *< ⌊*n *- 1⌋. The reverse bias occurs as *K *approaches 2*n − *2 from below.

To determine *q*(K*|*K*), we identify that our kernel chooses a *δ_k _*= 0 with probability (2*n − *2 *− K *)/(2*n − *2) and a *δ_k _*= 1 with probability *K/*(2*n − *2). Therefore, if K* = *K *+ 1, *q*(K*|*K*) is the former probability and if K* = *K *- 1, *q*(K*|*K*) is the latter. Forming the Hastings ratio

(10) $$\frac{q(K|K^{⋆})}{q(K^{⋆}|K)}=\{\begin{array}{ll} \frac{K+1}{2n-2-K} & \text{if}\;K^{⋆}=K+1,\\ \frac{2n-2-K+1}{K} & \text{if}\;\;K^{⋆}=K-1. \end{array}$$

This derivation provides an important lesson for those new to MCMC implementation; the Hastings ratio may vary depending on the model parameterization; it is, therefore, necessary to calculate the ratio as a function of the same parameterization as the prior.

In cases where the swap event relaxes the prior variance on the rate multiplier *ϕ_k_*, we simultaneously propose a new value for $\phi_{k}^{⋆}\neq 1$. We draw this value from the prior given in Equation (4).

Proposals involving changes to the tree topology are based on existing tree proposal moves in the BEAST software framework with a small modification to track the augmented data at the nodes [see Additional file 1].

#### Model selection

Statistical inference divides into two intertwined approaches: parameter estimation and model selection. For the former, parameter inference relies on empirical estimates of *P*(***θ|*Y**) that we tabulate from the MCMC draws. Model selection often represents a more formidable task. The natural selection criterion in a Bayesian framework is the Bayes factor [28-30]. The Bayes factor *B*_10 _in favor of ℳ_1 _over ℳ_0 _is the ratio of the marginal likelihoods of ℳ_1 _and ℳ_0_,

(11) $$B_{10}=\frac{P(Y|{ℳ}_{1})}{P(Y|{ℳ}_{0})}=\frac{P({ℳ}_{1}|Y)}{P({ℳ}_{0}|Y)}/\frac{P({ℳ}_{1})}{P({ℳ}_{0})},$$

and informs the phylogeneticist how she (he) should change her (his) prior belief *P*(ℳ_1_)/*P*(ℳ_0_) about the competing models in the face of the observed data. Involving the evaluation of two different normalizing constants, Bayes factors are often challenging to estimate.

By fortuitous construction, we side-step this computational limitation when estimating the Bayes factor in favor of a global clock (GC) model ℳ_GC _over the RLC model ℳ_RLC_. Model ℳ_GC _occurs when *K *= 0, conveniently nested within model ℳ_RLC_. Consequentially, the *P*(*K *= 0|ℳ_RLC_) equals the prior probability of ℳ_GC_, and *P*(*K *= 0|**Y**,ℳ_RLC_) yields *P*(ℳ_GC_|**Y**). Given this, a Bayes factor test of ℳ_GC _only requires simulation under the RLC model. The Bayes factor in favor of a global clock

(12) $$B_{\text{GC}}=\frac{P(K=0|Y,{ℳ}_{\text{RLC}})}{1-P(K=0|Y,{ℳ}_{\text{RLC}})}{\left(\frac{P(K=0|{ℳ}_{\text{RLC}})}{1-P(K=0|{ℳ}_{\text{RLC}})}\right)}^{-1}.$$

To calculate the ratio of marginal likelihoods we need only an estimator $\hat{\text{P}}$ of *P*(*K *=0|**Y**, ℳ_RLC_). The Ergodic Theorem suggests that we let

(13) $$\hat{\text{P}}=\sum_{\ell =1}^{L}1\left\{K^{\left(\ell \right)}=0\right\},$$

where 1{·} is the indicator function. Occasionally $\hat{\text{P}}$ becomes a poor estimator when *P*(*K *= 0|**Y**,ℳ_RLC_) decreases below ϵ or increases above 1 - ϵ for ϵ ≈ 1/L. In such situations, there are alternatives that depend on MCMC chains generated under several different prior probabilities *P*(*K *= 0|ℳ_RLC_) [31]. The Bayes factor then provides the mechanism to combine results from the multiple chains and to rescale back to a believable prior.

## Results

To explore the utility of the RLC model, we consider three well-studied examples that span the evolutionary scales from millions of years down to annual seasons. The first example investigates rate variation of several nuclear genes across the radiation of mammals [32]. Previous analyses fit these data under an unrooted phylogenetic model, and then rely on *post hoc *heuristics while conditioning on the maximum likelihood tree to identify local molecular clocks. We exploit the RLC model to simultaneously infer both the tree and locations of local clocks. We then turn our attention to mtDNA evolution within primates [33,34] and examine a subset of the original data in which multiple studies endorse a molecular clock [15,35,36] and demonstrate the ease in which one can formally test for a global clock via the RLC model. In both examples, the RLC model performs consistently with expectations. We conclude with a survey of the temporal patterns of rate variation in hemagglutinin gene evolution and uncover a signature of multiple epochs of increasing rate without specifying prior knowledge of their existence.

### Radiation of rodents and other mammals punctuated by local clocks

[32] investigate the existence of local molecular clocks during the radiation of mammals with an eye to reconciling molecular divergence dates with fossil evidence. In their study, [32] condition on a fixed evolutionary tree and perform multiple pair-wise or local rate heterogeneity tests to construct an *ad hoc *ensemble of clock models. We re-examine the same first and second codon positions of ADRA2B, IRBP and vWF nuclear genes (2422 alignment sites) from 42 mammals under the RLC model. Following [32], we assume the GTR model for nucleotide substitution with discrete-Γ site-to-site rate variation and ignore process heterogeneity across genes.

**Figure 1:**
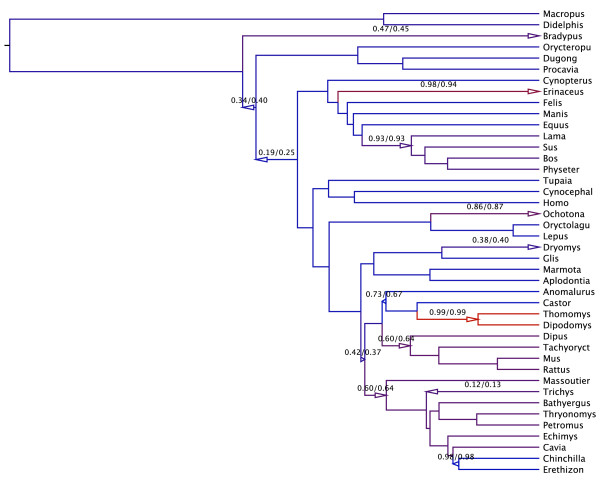
**Bayesian inference of random local clocks on mammalian data**. Most probable evolutionary tree relating three nuclear genes from 42 mammals [32]. The color of the branches in the tree indicate branch-specific relative rates from red (fast) to blue (slow). Regions with the same color signify local clocks. Branches with a posterior probability of a change in rate * >*0.1 are labeled with the estimated posterior probabilities from two independent runs. An arrow to the right signifies a rate increase on a branch (and its descendants), while an arrow to the left signifies a slow down.

Figure 1 presents the Bayesian consensus tree for these data. Major groupings persist across tree estimates; examples include the marsupial/placental divide and major placental clades. Small topological differences are not surprising given data uncertainty and that researchers inferred the original tree under an unrooted model whereas our estimate is based on a local-clock-constrained model of phylogenetic trees.

**Figure 2:**
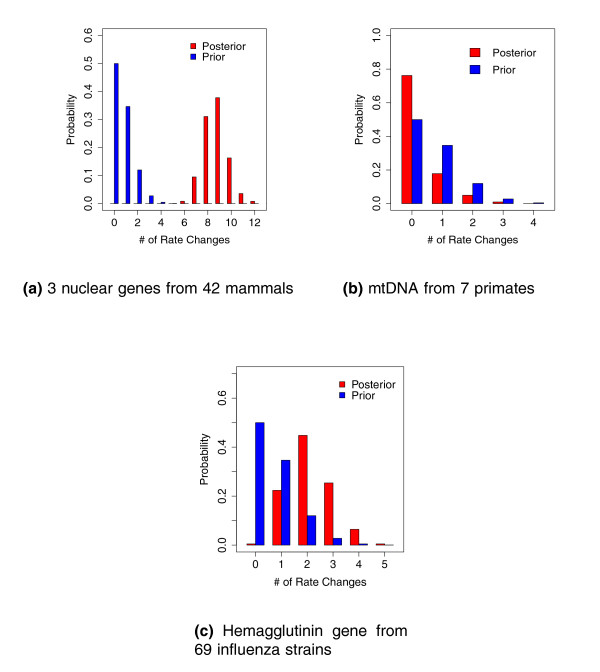
**Prior and posterior distributions of the number of rate changes for three molecular data sets**. Comparison of posterior (red) to prior (blue) probability mass functions of the number of rate changes *K *for the **(a) **mammal, **(b) **primate and **(c) **influenza examples. In all examples, the prior probability of a global molecular clock (*K *= 0) is 50%. Greater posterior than prior probability for *K *= 0 supports the global clock hypothesis (primates); while small or negligible posterior probability for *K *= 0 strongly rejects the hypothesis (mammals and influenza).

Amongst the very small collection of local clock models that [32] explore, they identity their best-fitting model as embracing five local clocks. This result matches surprisingly well with RLC model estimates that support between six to twelve local clocks (Figure 2(a)). Our estimate of the number of clocks integrates over all possible local clock assignments and trees and is naturally larger. We color branches in Figure 1 according to their branch-specific rates. Consistent with [32], the sloth (*Bradypus *), hedgehog (*Erinaceus *) and two geomyoid rodents (*Dipodomys *and *Thomomys *) exhibit higher rates of substitution. Comparing the posterior to prior probability that the number of rate changes *K *= 0 in Figure 2(a) clearly rejects a global clock within these data.

### Anthropoids' global clock

[33] and [34] present partial mtDNA sequences from nine primates, including two prosimians and seven anthropoids (monkeys/apes). The sequences comprise the protein coding regions for subunits 4 and 5 of the enzyme NADH-dehydrogenase and three tRNAs and contain 888 sites after the removal of alignment gaps. Since their publication, these data appear as molecular clock examples in several phylogenetic software releases [37-39]. [35,36] explore the strict molecular clock assumption in these data using a Bayesian approach and find good support for a clock among the anthropoids, but not between the anthropoids and prosimians, nor within the prosimians. The Bayes factor tests developed in these former studies require complicated calculations that lend themselves poorly to general use by evolutionary biologists. The RLC model provides a simple solution.

As an example in which a global clock should hold, we re-examine the seven anthropoids sequences under the RLC model. We employ the HKY85 [15] model for nucleotide substitution with discrete-Γ site-to-site rate variation. To keep exposition simple, we ignore structured rate heterogeneity between the concatenated genes and across codon position with genes; however, these important modeling aspects remain straight-forward to include and do not complicate the final Bayes factor calculations. To complete specification, we assume λ = log 2, such that there exists a 50% prior probability of a global clock.

**Figure 3:**
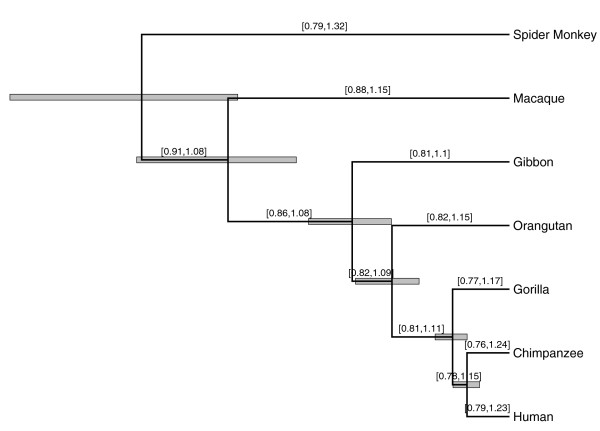
**Inferred mtDNA rates for primate phylogeny**. Most probable evolutionary tree relating seven mtDNA sequences from primates [33]. Gray boxed regions depict 95% Bayesian credible intervals (BCIs) for relative divergence times (that is, in units of expected substitutions per site). Recorded for all branches are their relative rate parameter *r_k _*95% BCIs. All intervals cover 1, suggesting little or no rate variation across the tree.

Figure 3 presents the *a posteriori *most probable tree relating these sequences. The topology of this tree recapitulates the current paradigm of primate evolution, including the nearest-neighbor relationship between humans and chimpanzees, for which these data originally helped settle [15,34]. We annotate the internal node heights in the figure with their posterior 95% Bayesian credible intervals (BCIs).

An important use of the molecular clock hypothesis is in estimating divergence times, and this ability remains under the RLC model. Near the tree branches in the figure, we also report 95% BCIs for the branch-specific relative rates *r_k_*. Notably, all intervals cover the global clock hypothesized value of 1, suggesting the existence of a global clock in these data. However, these intervals are univariate marginal reports of highly correlated random variables and multiple marginal assessments can lead to spurious conclusions. To test all branches simultaneously, we calculate *B*_GC _from knowledge of the model prior and an estimate $\hat{\text{P}}$of the posterior probability that number of rate changes *K *= 0. Figure 2(b) reports both the prior and estimate of the posterior probability mass function of *K*. A majority of the posterior mass falls on *K *= 0, even more so than the prior. From the figure, *B*_GC _= 3.3. While this Bayes factor is far from offering extreme support [28,29] for the global clock model itself, the balance of evidence favors a global clock over all other specific alternatives, and the global clock would be contained in any credible set of models.

### Temporal rate patterns in influenza

We examine hemagglutinin gene evolution from 69 strains of human influenza A [40]. These sequences represent serially sampled data; the earliest sequence stems from 1981 and the last from 1998, spanning a 17 year period. To infer the evolutionary tree and rate changes, we again employ the HKY85 model for nucleotide substitution, with Gamma-distributed rate heterogeneity among sites [24]. As priors, we assume an underlying coalescent process with a constant population size on the tree and a Poisson number of rate changes with an expected value of log2 [see Additional file 2, for an example BEAST 1.5.4 XML script]. This specification places 50% prior probability on the strict molecular clock hypothesis.

**Figure 4:**
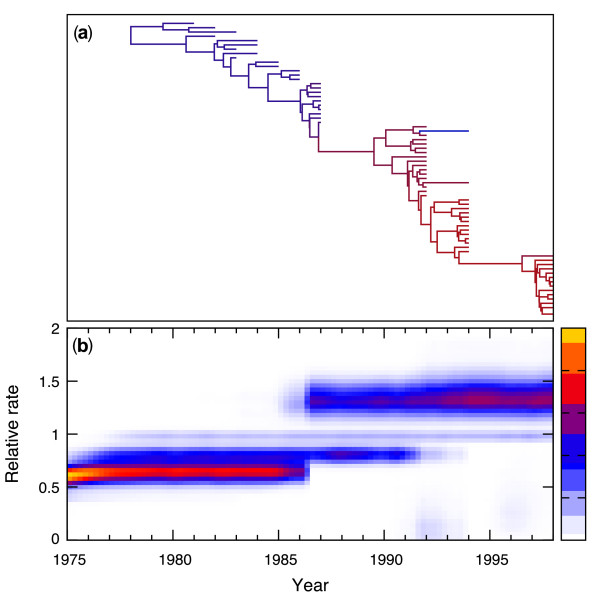
**Influenza A data analysis**. **(a) **Most probable evolutionary tree relating 69 hemagglutinin sequences from human influenza A. Branch coloring indicates inferred rates of nucleotide substitution, with blue denoting the slowest rates and red the fastest. **(b) **Rate heterogeneity of hemagglutinin sequence evolution over time. The plot traces the marginal distribution of relative substitution rates across time. White indicates low posterior density, and yellow/red indicates high density. The estimated rates are higher towards the present, with a notable jump in rate approximately six and ten years before the last sequence sample.

Figure 4(a) depicts the Bayesian consensus tree relating these sequences, along with posterior mean branch lengths scaled in real time. To examine rate variation, we color branches by their posterior mean relative rate of nucleotide substitution. Blue branches reflect the slowest rates of mutation through red branches that highlight regions of rapid change. From Figure 4(a), a general trend begins to take form of increasing rate variation over time; earlier branches to the left of the figure are mostly blue or purple, while late branches appear mostly red. We formally explore this trend in greater detail.

Figure 4(b) compares the posterior and prior mass functions relating the number of rate changes observed during hemagglutinin evolution. As expected from the observed variation in Figure 4(a), very little posterior mass falls on the existence of a global clock with zero rate changes. The modal number of rate changes is two. The Bayes factor rejecting a global clock is approximately 45, providing strong support [28,29].

Figure 4(a) examines the heterogeneity of rate variation as a process in time. To generate this plot, we discretize time before the last sequence sampling date into 92 bins (four per year). For each bin, we construct the empirical posterior density of relative rates active along the tree during that time-period. Rates that we color yellow or red occur with high posterior probability while rates proceeding towards white reflect lower probabilities. Consistent with the posterior mode of two rate changes shown in Figure 4(b), the two rate break-points in Figure 4(b) generate three distinct epochs in hemagglutinin evolution, with a trend towards increasing rates over time. The first epoch begins at the root of the evolutionary tree and continues until some point between 1986 and 1992. The final epoch concludes with the 1998 strains.

We caution against over-interpretation of the punctuated form of the transitions between epochs seen in Figure 4(b). While rate transitions may have arisen with such strong demarcation, their relative sharpness may be the result of the sampling pattern in this data set. The newer samples (between 1987 and 1998) are more densely sampled at each time point, while being separated by more time between samples (there are long temporal breaks in strain sampling between 1987 and 1992 and again between 1994 and 1998). Temporal changes in sampling pattern could be particularly problematic given the well accepted fact that the influenza virus population is subject to strong selection and the influenza data set used here has previously been shown to exhibit evidence for non-neutrality [40]. Richer taxon-sampling during the unsampled periods may clarify this issue, but remains beyond the scope of this methodological paper. Nonetheless, to confirm that the RLC model is performing appropriately, we do explore the temporal rate variation process in further detail using an explicitly temporal model of rate change. To do so these data were analyzed under a Bayesian implementation (Andrew Rambaut *pers comms*) of a fixed-epoch model [41]. The result reinforces the conclusion that these data do exhibit temporal rate variation [see Additional file 1 for analysis summary and Additional file 3 for the BEAST XML]. However, the fixed-epoch model requires *a priori *specification of the number of different rate-epochs on which to fit the data, and assumes each rate change occurs simultaneously across all lineages, whereas the RLC assumes no such prior knowledge.

## Discussion

Although it has been clear for quite some time that no universal molecular clock exists, a new question is emerging about what is the phylogenetic footprint of local molecular clocks. With increasing densely sampled phylogenetic trees, we should start to be able to get estimates of the extent of local clocks.

A major limitation of local clock models has been a dearth of methods to appraise all the possible rate assignments for various lineages [42]. BSSVS permits the efficient exploration of all 2^2*n-*2 ^possible local clock models and automatically returns the most parsimonious descriptions of the data.

The RLC description finds notable similarity to a compound Poisson process for rate variation [4]. Under this process, a Poisson number of change-points fall independently onto the branches of a phylogenetic tree. At each change-point, a Gamma-distributed random variable punctuates the current substitution rate. Without additional external information, the number of change-points (if greater than 1) and their specific locations along the branch are not identifiable by the likelihood, though this can be resolved by the prior. However this lack of identifiability places into question the benefit of allowing the large (in fact infinite) augmented state space of change points in the compound Poisson process that our BSSVS approach avoids. Under BSSVS, either there exists no change along a branch or there exists more than one and the new branch-specific rate represents an average over all events and their locations. BSSVS can also generalize to model heterogeneity in aspects of the CTMC process beyond rate variation. Examples we are considering include random local changes in nucleotide composition; a natural extension of previous work on modeling compositional heterogeneity [43]. It is also possible to use this approach to model random local changes in parameters of the tree prior [44].

Compared to the auto-correlated rate models [3], the RLC approach imparts some different prior assumptions on rate variance among branches. For example, the prior variance on a lineage-specific rate depends on the number of internal nodes traversed between the root and branch, not the time-duration. Obviously, this feature vanishes as the marginal prior on rates integrates over all possible trees. In the RLC model the number of traversed nodes reflects the number of sampled speciation events a lineage has encountered. The evolutionary and sampling scenarios for which this serves as a better proxy for rate change than does time-duration is outside the scope of this work. Formal model testing can help settle this debate on a dataset-by-dataset basis. We have not attempted model comparison between the RLC and other relaxed clock models as part of this work, as it is a very challenging task. New methods for computing Bayes factors between non-nested phylogenetic models, such as path sampling [45,46] and stepping-stone sampling [47] may improve this situation in the future.

Further, hybrid models remain within reach in which rate multipliers ***ϕ ***draw *a priori *from a multivariate distribution. The multivariate generalization of the Gamma is a Wishart, characterized by a scale matrix. This scale matrix could be a function of the time-tree.

While the transition kernels we employ in this paper successfully explore the posterior distribution for the three examples, we can envision datasets for which our algorithm would have difficulties producing accurate estimates of the posterior distribution. High correlation most likely exists between the evolutionary tree ***τ ***and location indicators ***δ ***along ***τ ***at which local clocks start. Some datasets may possess posterior support for alternative trees whose clock structures vary considerably. This situation poses a significant difficulty for our current transition kernels. These kernels alternate between updating ***τ ***with only small changes ***δ ***and updating ***δ ***conditional on ***τ***. In this construction, very rarely is it possible to make large moves in both tree-space and clock structures simultaneously, leading to potentially long mixing times. To remedy this, kernels that propose larger simultaneous jumps are warranted. While we are currently exploring different choices, finding kernels whose Hastings ratio remains convenient to calculate and function well across a range of datasets is proving challenging. We do, however, remain optimistic.

Alternatively, [48] encourages a collapsed Gibbs sampler via parameter marginalization when encountering high correlation. While it is computationally intractable to analytically integrate the model sampling density over all possible ***τ ***or all possible ***δ***, a "local" collapse suggests a viable option. [49] exploit such an approach when sampling over the joint space of trees and sequence alignments; when proposing an update to ***τ ***, these authors integrate over the smaller portion of alignment-space affected by jumping from the current to proposed tree; then, given the new tree, re-sample a consistent and probable alignment. For the RLC model, a "local" collapse equates to integrating out the location indicators *δ_k _*on branches near the affected portions of ***τ ***and reduces to a discrete summation over a modest number of combinations. There still exists correlation between indicators ***δ ***and rate multiplier ***ϕ***; however, we believe this correlation strength is much smaller than between that above, as the multipliers only enter into the likelihood when *δ_k _*= 1 and, hence, have considerably more freedom in their realized values. In any case, researchers should not blindly apply Bayesian samplers to new datasets; samplers require care and thought to ensure adequate exploration of the posterior parameter space.

## Conclusions

We have proposed an efficient method to sample over random local molecular clocks while simultaneously estimating the phylogeny. The new method conveniently allows a comparison of the strict molecular clock against a large array of alternative local molecular clock models. We have illustrated the method's utility on three example data sets involving mammal, primate and influenza evolution. We also explored a method to visualize the complex posterior distribution on the influenza data set which led to discovery of a strong temporal signal for the evolutionary rate in that data set, although this observation may well be attributed to temporal variation in sampling pattern. The examples that we have investigated suggest that large sequence datasets may only require a relatively small number of local molecular clocks to reconcile their branch lengths with a time scale. All of the analyses described here are implemented in the open access software package BEAST 1.5.4 http://beast-mcmc.googlecode.com/.

## Authors' contributions

Both authors developed the idea and conducted the main experiments. AJD implemented the Bayesian stochastic search variable selection in the BEAST 1.5 and BEAST 2 open source software packages. Both authors debugged the software and wrote supporting software to analyze and visualize the results. Both authors were involved in the writing of the manuscript.

## Supplementary Material

Additional file 1 **Supplementary Information**. This is a PDF file describing some additional details of the described methods including (i) a description of the proposal distribution for trees used in the RLC model and (ii) a summary of the analysis of the influenza data using a "fixed epoch" model that allows the rate of evolution to change at a specific time in the past. Click here for file

Additional file 2 **Human.H3.81-98_-_local_-_gamma.xml**. This is a BEAST XML input file compatible with BEAST 1.5.4 that implements the model combination used to analyze the influenza data set under the RLC model. Click here for file

Additional file 3 **Human.H3.81-98_-_2rate.xml**. This is a BEAST XML input file compatible with BEAST 1.5.4 that implements the "fixed epoch" model used to confirm the signal for a temporal ate change in the influenza data set. Click here for file

## References

[B1] Sarich VM Wilson AC Immunological time scale for hominid evolution Science 1967 158 1200 1203 10.1126/science.158.3805.1200 4964406

[B2] Zuckerkandl E Pauling L Evolutionary Divergence and Convergence in Proteins 1965 New York: Academic Press 97 166

[B3] Thorne JL Kishino H Painter IS Estimating the rate of evolution of the rate of molecular evolution Mol Biol Evol 1998 15 1647 1657 9866200 10.1093/oxfordjournals.molbev.a025892

[B4] Huelsenbeck JP Larget B Swofford D A compound poisson process for relaxing the molecular clock Genetics 2000 154 1879 1892 10747076 10.1093/genetics/154.4.1879 PMC1461022

[B5] Sanderson MJ Estimating absolute rates of molecular evolution and divergence times: a penalized likelihood approach Mol Biol Evol 2002 19 101 109 11752195 10.1093/oxfordjournals.molbev.a003974

[B6] Drummond AJ Ho SYW Phillips MJ Rambaut A Relaxed phylogenetics and dating with confidence PLoS Biol 2006 4 e88 10.1371/journal.pbio.0040088 16683862 PMC1395354

[B7] Rannala B Yang Z Inferring speciation times under an episodic molecular clock Syst Biol 2007 56 453 466 10.1080/10635150701420643 17558967

[B8] Gillespie JH Lineage effects and the index of dispersion of molecular evolution Mol Biol Evol 1989 6 636 647 2488476 10.1093/oxfordjournals.molbev.a040576

[B9] Gillespie JH The Causes of Molecular Evolution 1991 Oxford: Oxford University Press

[B10] Bromham L Penny D The modern molecular clock Nat Rev Genet 2003 4 216 224 10.1038/nrg1020 12610526

[B11] Yoder AD Yang Z Estimation of primate speciation dates using local molecular clocks Mol Biol Evol 2000 17 1081 1090 10889221 10.1093/oxfordjournals.molbev.a026389

[B12] Drummond AJ Rambaut A BEAST: Bayesian evolutionary analysis by sampling trees BMC Evol Biol 2007 7 214 10.1186/1471-2148-7-214 17996036 PMC2247476

[B13] Felsenstein J Inferring Phylogenies 2004 Sunderland, MA: Sinauer Associates, Inc

[B14] Lange K Applied Probability 2003 New York: Springer [Springer Texts in Statistics.]

[B15] Hasegawa M Kishino H Yano T Dating the human-ape splitting by a molecular clock of mitochondrial DNA J Mol Evol 1985 22 160 174 10.1007/BF02101694 3934395

[B16] Lanave C Preparata G Saccone C Serio G A new method for calculating evolutionary substitution rates J Mol Evol 1984 20 86 93 10.1007/BF02101990 6429346

[B17] Yang Z Among-site rate variation and its impact on phylogenetic analyses Trends Ecol Evol 1996 11 367 372 10.1016/0169-5347(96)10041-0 21237881

[B18] Felsenstein J Evolutionary trees from DNA sequences: a maximum likelihood approach J Mol Evol 1981 17 368 376 10.1007/BF01734359 7288891

[B19] Kishino H Thorne JL Bruno WJ Performance of a divergence time estimation method under a probabilistic model of rate evolution Mol Biol Evol 2001 18 352 361 11230536 10.1093/oxfordjournals.molbev.a003811

[B20] Thorne JL Kishino H Divergence time and evolutionary rate estimation with multilocus data Syst Biol 2002 51 689 702 10.1080/10635150290102456 12396584

[B21] George EL McCulloch RE Variable selection via Gibbs sampling J Am Stat Assoc 1993 88 881 889 10.2307/2290777

[B22] Kuo L Mallick B Variable selection for regression models Sankhya B 1998 60 65 81

[B23] Chipman H George EI McCulloch RE The practical implementation of Bayesian model selection Model Selection 2001 38 Benchwood, OH: Institute of Mathematical Statistics 67 134 [*IMS Lecture Notes - Monograph Series*]

[B24] Yang Z Maximum likelihood phylogenetic estimation from DNA sequences with variable rates over sites: approximate methods J Mol Evol 1994 39 306 314 10.1007/BF00160154 7932792

[B25] Metropolis N Rosenbluth AW Rosenbluth MN Teller AH Teller E Equations of state calculations by fast computing machines J Chem Phys 1953 21 1087 1092 10.1063/1.1699114

[B26] Hastings WK Monte Carlo sampling methods using Markov chains and their applications Biometrika 1970 57 97 109 10.1093/biomet/57.1.97

[B27] Drummond AJ Nicholls GK Rodrigo AG Solomon W Estimating mutation parameters, population history and genealogy simultaneously from temporally spaced sequence data Genetics 2002 161 1307 1320 12136032 10.1093/genetics/161.3.1307 PMC1462188

[B28] Jeffreys H Some tests of significance, treated by the theory of probability Proc Camb Philos Soc 1935 31 203 222 10.1017/S030500410001330X

[B29] Jeffreys H Theory of Probability 1961 1 London: Oxford University Press

[B30] Kass RE Raftery AE Bayes factors J Am Stat Assoc 1995 90 773 795 10.2307/2291091

[B31] Suchard MA Weiss RE Sinsheimer JS Models for estimating Bayes factors with applications in phylogeny and tests of monophyly Biometrics 2005 61 665 673 10.1111/j.1541-0420.2005.00352.x 16135017

[B32] Douzery EJP Delsuc P Stanhope MJ Huchon D Local molecular clocks in three nuclear genes: divergence times for rodents and other mammals and incompatibility among fossil calibrations J Mol Evol 2003 57 S201 S213 10.1007/s00239-003-0028-x 15008417

[B33] Brown WM Prager EM Wang A Wilson AC Mitochondrial DNA sequences of primates, tempo and mode of evolution J Mol Evol 1982 18 225 239 10.1007/BF01734101 6284948

[B34] Hayasaka K Gojobori KT Horai S Molecular phylogeny and evolution of primate mitochondrial DNA Mol Biol Evol 1988 5 626 644 3146681 10.1093/oxfordjournals.molbev.a040524

[B35] Suchard MA Weiss RE Sinsheimer JS Bayesian selection of continuous-time Markov chain evolutionary models Mol Biol Evol 2001 18 1001 1013 11371589 10.1093/oxfordjournals.molbev.a003872

[B36] Suchard MA Weiss RE Sinsheimer JS Testing a molecular clock without an outgroup: derivations of induced priors on branch length restrictions in a Bayesian framework Syst Biol 2003 52 48 54 10.1080/10635150390132713 12554439

[B37] Larget B Simon DL Markov chain Monte Carlo algorithms for the Bayesian analysis of phylogenetic trees Mol Biol Evol 1999 16 750 759

[B38] Huelsenbeck JP Ronquist F Mrbayes: Bayesian inference of phylogenetic trees Bioinformatics 2001 17 754 755 10.1093/bioinformatics/17.8.754 11524383

[B39] Yang Z PAML 4: a program package for phylogenetic analysis by maximum likelihood Mol Biol Evol 2007 24 1586 1591 10.1093/molbev/msm088 17483113

[B40] Drummond AJ Suchard MA Fully Bayesian tests of neutrality using genealogical summary statistics BMC Genet 2008 9 68 10.1186/1471-2156-9-68 18976476 PMC2645432

[B41] Drummond A Forsberg R Rodrigo AG The inference of stepwise changes in substitution rates using serial sequence samples Mol Biol Evol 2001 18 1365 1371 11420374 10.1093/oxfordjournals.molbev.a003920

[B42] Sanderson MJ A nonparametric approach to estimating divergence times in the absence of rate consistency Mol Biol Evol 1997 14 1218 1231

[B43] Foster PG Modeling compositional heterogeneity Syst Biol 2004 53 485 495 10.1080/10635150490445779 15503675

[B44] Gray RD Drummond AJ Greenhill SJ Language phylogenies reveal expansion pulses and pauses in pacific settlement Science 2009 323 479 483 10.1126/science.1166858 19164742

[B45] Lartillot N Philippe H Computing Bayes factors using thermodynamic integration Syst Biol 2006 55 195 207 10.1080/10635150500433722 16522570

[B46] Beerli P Palczewski M Unified framework to evaluate panmixia and migration direction among multiple sampling locations Genetics 2010 185 313 326 10.1534/genetics.109.112532 20176979 PMC2870966

[B47] Fan Y Wu R Chen M-H Kuo L Lewis PO Choosing among partition models in Bayesian phylogenetics Mol Biol Evol 2010 in press 20801907 10.1093/molbev/msq224 PMC3002242

[B48] Liu JS The collasped Gibbs sampler in Bayesian computations with applications to a gene regulation problem J Am Stat Assoc 1994 89 958 966 10.2307/2290921

[B49] Redelings BD Suchard MA Joint Bayesian estimation of alignment and phylogeny Syst Biol 2005 54 401 418 10.1080/10635150590947041 16012107

