# Supplementary material for: Bayesian random local clocks, or one rate to rule them all
Source: BMC Biol. 2010 Aug 31;8:114. doi: 10.1186/1741-7007-8-114 (PMC2949620; doi:10.1186/1741-7007-8-114)
Supplement: Additional file 1 — Supplementary Information. This is a PDF file describing some additional details of the described methods including (i) a description of the proposal distribution for trees used in the RLC model and (ii) a summary of the analysis of the influenza data using a "fixed epoch" model that allows the rate of evolution to change at a specific time in the past. [file 1741-7007-8-114-S1.pdf]

Supplementary information for: Bayesian random local clocks, or one rate to rule them all

Alexei J Drummond, Marc A Suchard

1 Tree proposal in the Random Local Clocks model

Figure 1 shows the application of two successive Fixed Node Prune and Regraft [1] tree proposals, illustrating that the indicator parameter (and its corresponding rate parameter ϕ) "travel" with the node during the tree proposal move. This is also true of other tree proposals that effectively move a single node (and its subtree), such as the Narrow Exchange [2]. For tree moves involving the movement of two subtrees (such as the Wide Exchange [2]) both pairs of indicator/rate parameters at the two roots of the pruned subtrees "travel" with their respective nodes.

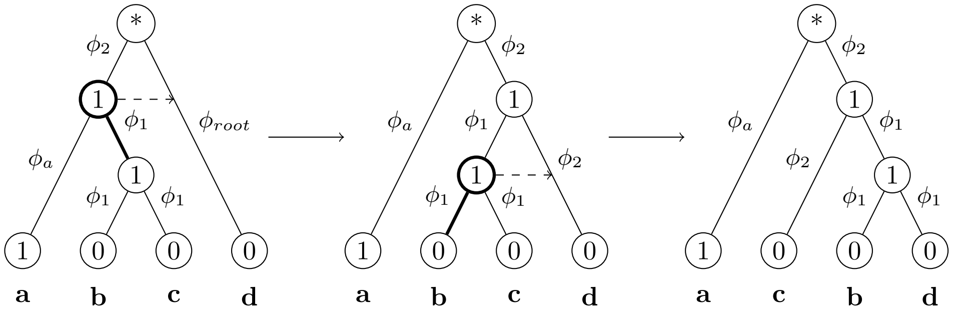

Figure 1: Two successive tree moves showing that the indicator variable "travels" with the node that it labels. The corresponding rate parameter (ϕ) also stays with the node, and so the branch above the new location of the node is subject to a new rate, *if* the indicator variable is 1. The branches of the trees are labelled with the rate parameter that applies to them.

2 Bayesian estimation of stepwise change in substitution rate

We used a Bayesian implementation (Andrew Rambaut, *pers. comm.*) of a stepwise-change model of substitution rate [3] to confirm the increase in estimated rate observed in the application of the Random Local Clock (RLC) model to an influenza data set. The time tree was partitioned into branch segments before and after 13 years from the most recent sample. Each of the two sets of branch segments were assigned a possible different substitution rate that were estimated by Bayesian MCMC.

The marginal estimates of the two rates were 3.1 (2.0 – 4.4) $\times 10^{-3}$ and 5.7 (4.7 – 6.7) $\times 10^{-3}$. The 95% highest posterior density intervals do not overlap, and thus strongly suggest a significant difference in the rate on either side of the dividing line.

Figure 2 shows a heat plot for the marginal posterior distribution of the average rate through time under this model.

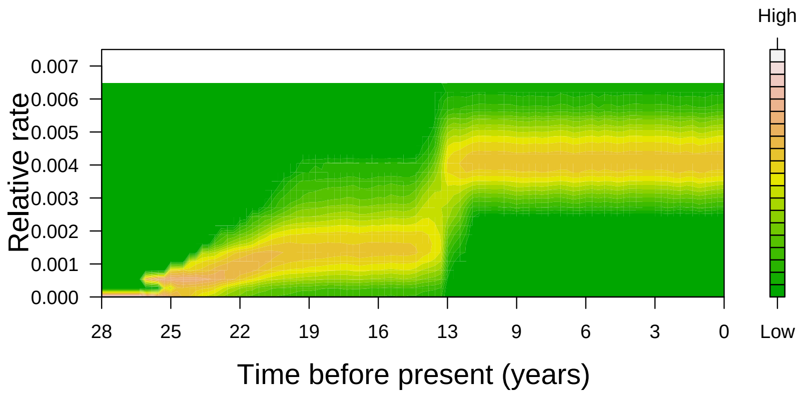

Figure 2: The posterior distribution of the average rate across all lineages through time under a model in which there are two substitution rates, one for all branch segments older than 13 years before the first sample, and one for all segments younger than 13 years before the most recent sample.

References

[1] Hohna S, Defoin-Platel M, Drummond A: **Clock-constrained tree proposal operators in Bayesian phylogenetic inference**. In *8th IEEE International Conference on BioInformatics and BioEngineering, 2008. BIBE 2008* 2008:1–7.

[2] Drummond A, Nicholls G, Rodrigo A, Solomon W: **Estimating mutation parameters, population history and genealogy simultaneously from temporally spaced sequence data**. *Genetics* 2002, **161**:1307–1320.

[3] Drummond A, Forsberg R, Rodrigo A: **The inference of stepwise changes in substitution rates using serial sequence samples**. *Molecular Biology and Evolution* 2001, **18**:1365–1371.
